# Adrenal Steroids Uniquely Influence Sexual Motivation Behavior in Male Rats

**DOI:** 10.3390/bs2030195

**Published:** 2012-08-31

**Authors:** George T. Taylor, Joshua T. Dearborn, Susan E. Maloney

**Affiliations:** 1 Behavioral Neuroscience Group, University of Missouri-St. Louis, 8001 Natural Bridge Road, St. Louis, MO 63110, USA; E-Mails: geot@umsl.edu (G.T.T.); sem885@mail.umsl.edu (S.E.M.); 2 Department of Psychiatry, School of Medicine, Washington University in St. Louis, Box 8134, 660 S. Euclid, St. Louis, MO 63110, USA

**Keywords:** DHEA, androstenedione, corticosteroids, motivation, mechanisms

## Abstract

The androgenic adrenal steroids dehydroepiandrosterone (DHEA) and 4α-androstenedione (4-A) have significant biological activity, but it is unclear if the behavioral effects are unique or only reflections of the effects of testosterone (TS). Gonadally intact male Long-Evans rats were assigned to groups to receive supplements of DHEA, 4-A, TS, corticosteroid (CORT), all at 400 µg steroid/kg of body weight, or vehicle only for 5 weeks. All males were tested in a paradigm for sexual motivation that measures time and urinary marks near an inaccessible receptive female. It was found that DHEA and 4-A supplements failed to influence time near the estrous female in the same way TS supplements did, and, indeed, 5 weeks of 4-A administration reduced the time similar to the suppressive effects of CORT after 3 weeks. Further, animals treated with DHEA or 4-A left fewer urinary marks near an estrous female than TS and control groups. These results suggest that DHEA and 4-A are not merely precursors of sex hormones, and provide support for these steroids influencing the brain and behavior in a unique fashion that is dissimilar from the effects of TS on male sexual behavior.

## 1. Introduction

The androgenic adrenal steroids dehydroepiandrosterone (DHEA) and 4α-androstenedione (4-A) have significant biological activity, yet the nature of their significance, especially in the brain, remains uncertain. Similarly, these steroids’ direct effects on behavior have yet to be thoroughly explained, in contrast to the extensive research on the behavioral consequences of the androgen testosterone (TS), a potent sex steroid. Testosterone is used as a medical treatment for many conditions, most prominently sexual dysfunction. Illicit use to enhance athletic performance, particularly among men and boys, has been widespread since the 1980s [1]. Effects of similar use of DHEA and 4-A have not yet been adequately elucidated. Because these adrenal steroids are precursors of TS it remains possible, as was originally suspected, that their effects will be similar, if not identical, to those of TS. In this view, DHEA and 4-A are simply substrates for the sex steroids which then initiate activity by binding the androgen receptor (TS and dihydrotestosterone (DHT)) or estrogen receptors (estradiol (E2)). 

Still, there remains the possibility that DHEA and 4-A have unique impacts on complex behaviors, something that has been difficult to demonstrate. One approach is to employ a behavior that is highly sensitive to the sex steroids. Testosterone and its metabolites play a key role in both male and female interest in the opposite sex [2]. Using a commonly-used measure of sexual motivation in male rats, choosing between receptive and non-receptive females, it would seem possible to establish effects of the adrenal products. 

The present experiment was designed to provide supplements of DHEA and 4-A and assess sexual motivation in intact male rats. Comparison conditions included supplements of either TS or corticosterone (CORT), presumably to be upper and lower limits of sexual motivation. CORT is included because there is substantial evidence that the hypothalamic-pituitary-adrenal axis (HPA) suppresses the hypothalamic-pituitary-gonadal (HPG) axis [3,4]. The vehicle-only group is to serve as a comparison for no effect, *i.e*., if a supplement group is similar to the vehicle group, it is concluded that the supplement had no effect on behavior. The TS-supplemented group is expected to represent the upper limit of sexual motivation, but also to serve as a measure of comparison for the DHEA and 4-A groups.

The logic is that, if DHEA and 4-A serve only as precursors to TS, those males will behave similarly to the TS animals, *i.e*., spending more time and urinary marking more near the receptive female. This would lend support to the hypothesis that these steroids merely affect behavior because they are likely metabolized into TS; they only impart effects after conversion, and therefore result in behavior similar to supplemental TS. If DHEA and 4-A animals behave more similarly to non-supplemented controls or even CORT animals, the support accrues to the androgenic adrenal steroids having unique effects on brain and behavior.

## 2. Results and Discussion

All variables were evaluated for normality previous to multivariate analysis; no transformations were necessary. Four outliers were revealed in the open field data (*Z* ≥ 2.5) and each was replaced with the mean for that variable. 

### 2.1. Sexual Motivation

**Figure 1:**
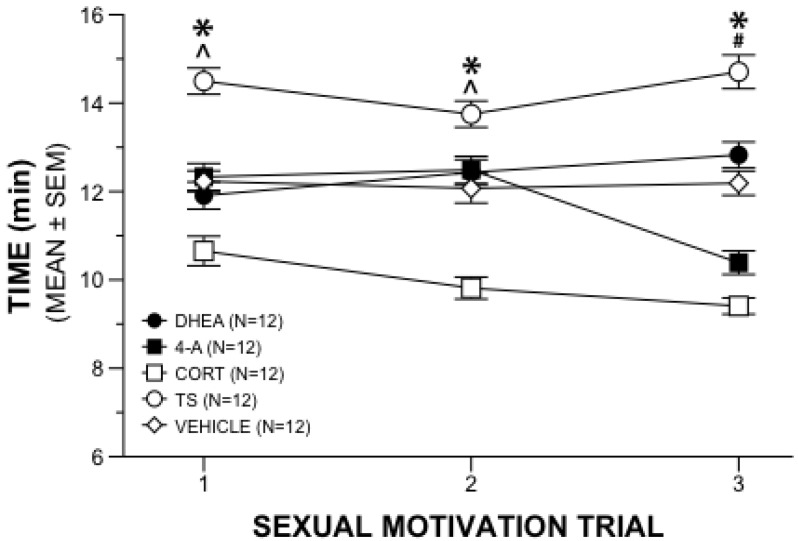
Sexual Motivation Behavior. Mean (±SEM) time gonadally intact male rats given chronic steroid or vehicle supplement spent near estrous female during three weeks of 20-min sexual motivation trials. (*) TS significantly different from all other groups; (^) CORT significantly different from all other groups; (#) CORT significantly different from all other groups except 4-A.

A repeated measures 5 × 3 ANOVA on the time near the receptive female in the sexual motivation paradigm revealed a significant interaction of drug and week (*F*_8,110_ = 9.336, *p* = 0.001). Further analyses with main effects allowed comparisons between treatment groups at each week of testing and within treatment groups across the three weeks. Between group analyses revealed time differences among the hormone treatments during the first week of sexual motivation testing (*F*_4,55_ = 21.700, *p* = 0.001). Subsequent comparisons with Tukey’s HSD tests (*p* < 0.05) indicated that males administered TS spent more time near the estrous female than the other males, and males administered CORT spent less time near the estrous female than all other groups (Figure 1). Androstenedione and DHEA did not differ from vehicle during the first week. 

Those differences were mirrored in the second week of testing (*F*_4,55_ = 23.494, *p* = 0.001) in that TS animals spent more time near the estrous female, and the CORT animals spent less time near the estrous female, than all other groups. Comparisons of drug treatment at the third week of sexual motivation testing (*F*_4,55_ = 52.735, *p* = 0.001) revealed that males administered TS continued to spend more time near the estrous female than all other groups. The CORT group spent less time near the estrous female than other groups except for the 4-A animals. There were no differences between CORT and 4-A during the third week.

Within-group main effects provided a snapshot of changes in time near the receptive female over the 3 weeks of sexual motivation testing. Results were that, whereas the TS rats showed increased motivation, each of the three adrenal supplement groups were less interested in the receptive females over weeks. Specifically, animals administered 4-A (*F*_2,54_ = 23.876, *p* = 0.001) spent less time near the estrous female in week 3 compared to both weeks 1 and 2. Males administered DHEA (*F*_2,54_ = 3.515, *p* = 0.037) spent more time with the estrous female during week 3 when compared with week 1, but not week 2. Males administered CORT (*F*_2,54_ = 6.740, *p* = 0.002) spent less time near the estrous female during weeks 2 and 3 compared to week 1. Males receiving TS supplements (*F*_2,54_ = 5.036, *p* = 0.010) spent more time near the estrous female in week 3 when compared to week 2, although not to week 1. Vehicle control animals did not differ in this marker of sexual motivation across the three weeks of observation.

### 2.2. Urinary Marking and Open Field Activity

**Figure 2:**
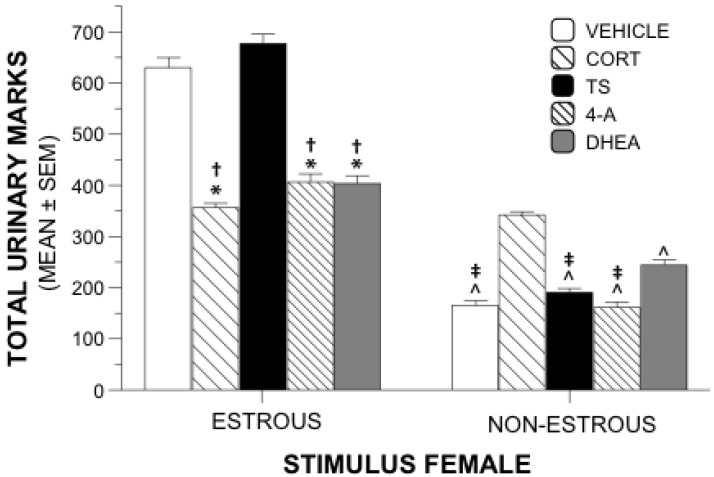
Urinary Marking Behavior. Mean (±SEM) total urinary marks near stimulus females during sexual motivation testing. Groups of males were gonadally intact and were administered supplements of steroid or vehicle during 3 weeks of testing. (*) Significantly different from TS; (†) Significantly different from vehicle; (^) Significantly different from CORT; (‡); Significantly different from DHEA.

Total numbers of urinary marks near the estrous female or the nonestrous female were used as an additional measure of sexual motivation. A 5 × 2 repeated measures ANOVA revealed a significant interaction of drug and urinary marks (*F*_4,55_ = 145.26, *p* = 0.001). Main effects allowed comparisons between treatment groups of total marks near each stimulus female. Between-group analyses showed significant differences of total urinary marks near the estrous female (*F*_4.55_ = 87.687, *p* = 0.001). Post hoc tests using Tukey’s HSD (*p* < 0.05) showed that animals receiving TS or vehicle marked significantly more near the estrous female than those animals receiving 4-A, DHEA, or CORT (Figure 2). There was no difference in urinary marking between the vehicle and TS treatment groups, and the markings were similar among the 4-A, DHEA, and CORT groups.

Between-group differences were also significant for total urinary marks near the nonestrous female (*F*_4,55_ = 78.022, *p* = 0.001). Tukey’s post hoc analysis revealed that the CORT animals marked more near the nonestrous female than all other groups. DHEA-treated animals marked near the nonestrous female significantly more than 4-A-, vehicle-, and TS- animals. Numbers of marks near the non-estrous female were similar among the 4-A, TS, and vehicle groups.

Within-group main effects revealed significant differences between marks near the estrous female and the nonestrous female within each treatment group (*F*_4,55_ = 39.134, *p* = 0.001). Males treated with 4-A, DHEA, TS, or vehicle marked significantly more near the estrous female than the nonestrous female (*p* = 0.001 for all comparisons). CORT-treated animals marked similarly near both stimulus females (*p* = 0.401).

Numbers of lines crossed in the open field were totaled across the three weeks of testing. A univariate ANOVA revealed no significant differences among groups (*F*_4,55_ = 0.654, *p* = 0.627).

### 2.3. Discussion

Results of the experiment revealed that supplements of 4-A or DHEA did not mimic the effects of TS supplements on sexual motivation in intact male rats. Both groups were significantly less interested than the TS supplemented group in a receptive female in each of three test sessions. Further, during the third week of testing, the 4-A animals showed similarly suppressed interest as the CORT group in remaining near the female. Examination of an additional marker of sexual motivation, urinary marking near the estrous female, over all weeks indicated that DHEA and 4-A marked similarly to the CORT animals and significantly less than both TS and vehicle control animals.

The present study is part of a research program on adrenal-testicular steroidal interactions using both castrated and intact males [5,6]. Here we used intact animals partly because of recent promotions of testosterone supplements in men experiencing perceived or actual low libido. Also, previous findings [7,8,9] indicating that TS supplements can increase sexual motivation in intact rats provided a segue to the empirical question posed for the experiment. That is, will the adrenal precursors androstenedione and dehydroepiandrosterone induce a similar pattern of behavioral changes as TS? The results would also address the conceptual question of whether 4-A and DHEA serve only as precursors to TS or do the adrenal steroids have their own individual influence on brain and behavior. Inclusion of males receiving CORT supplements provided a control group for an adrenal steroid that often is found to suppress sexual behavior.

Findings from the experiment support the hypothesis that DHEA and 4-A have direct influences on behavior, and that they are not simply destined to influence function in an indirect manner by conversion to TS, E2, or DHT. If the adrenal steroids produced behavioral results similar to the TS group, it could be reasonably concluded that DHEA and 4-A lead to behavioral changes only after conversion to TS (indirect) and are without direct influences. Results from this experiment show that this is unlikely to be the case; DHEA and 4-A produced a behavioral footprint different than that of TS, indicating that they likely affect behavior directly and without requiring metabolic conversion. In agreement with previous findings [10,11] that increased levels of the stress hormone CORT accompany attenuated male sexual behavior, CORT-supplemented males represented a basement influence on sexual motivation. Unsurprisingly, TS treatment provided a “ceiling effect” for the measures of sexual motivation, resulting in the highest levels of anticipatory behavior [12]. These findings also suggest that whether or not TS supplements suppressed testicular activity, they enhanced a TS-sensitive behavior [8,13].Results from the 4-A and DHEA treatment groups did not mimic this effect and in one instance were actually similar to CORT, providing support for 4-A and DHEA acting directly on the brain and sexual behavior. Decreased urine marking behavior indicates that DHEA and 4-A have their own direct suppressive effect on TS-like behaviors. The results also indicate that DHEA and 4-A are biologically active in ways that significantly affect behavior, indicating that any differences from TS are not attributable to the two simply having no effects, *i.e.*, replicating the control group.

An important consideration for the different behavioral results yielded from TS, 4-A, and DHEA treatment is the complex metabolic cascade of the steroids. Our recent review [14] outlined the various possible pathways a steroid precursor can follow. One outcome is adrenal steroid conversion to TS, but another is diversion to estrogens. DHEA is converted to 4-A, which then can be metabolized into either TS or estrone (E1). If converted into E1, the next step in the metabolic cascade is estradiol. Further, TS itself serves as a direct precursor to E2. Therefore while it is entirely plausible that 4-A or DHEA supplement would result in increased TS and androgen-sensitive behaviors, it is equally plausible that such treatment would result in increased E2. The possibility of this secondary pathway to the estrogens, however, cannot explain our results. Both E1 and E2 bind the estrogen receptor (ER) in the periphery and the brain, and binding of the ER is likely one mechanism for male sexual behavior, including motivation [12]. The estrogen receptor is also a likely mechanism for male urine marking behavior [15]. Whether converted to TS or E2, the results would mimic TS supplements if DHEA and 4-A served as mere precursors. This is of particular interest when interpreting the results that chronic 4-A supplements suppress sexual motivation compared to the control group. Conversion to TS or E2 would likely lead to increased sexual motivation behavior, but chronic 4-A decreased motivation in the 4-A group of intact males. Although previous studies using various species, including rats, cite the ability of 4-A to reinstate sexual behavior following castration [16,17,18,19] there is little, if any, research explaining the effects of 4-A supplements administered to intact males. Our data indicate the possibility of a feedback, or down-regulating, mechanism following chronic 4-A treatment that has not been explained previously.

A concern for all hormone research is the issue of acute *versus* chronic exposure. This is made apparent in the differences between the CORT-treated group presented here and that of a previous study [20]. Males supplemented with CORT in the present study experienced suppressed sexual motivation compared to controls as early as 3 weeks into exposure. We cannot say how early this difference could be detected in our animals since we began testing at 3 weeks of exposure, but Retana-Marquez and colleagues indicate in their 1998 study that it would likely be after 8 days of exposure. Interestingly, in their study CORT was administered at a fixed dose (2 mg) higher than we used, yet after 8 days of exposure intact male rats did not experience suppressed sexual behavior. It could be speculated, therefore, that even a high dose of CORT supplement must be administered for longer than 8 days to affect sexual behavior. Future studies like ours may consider behavioral testing at multiple time points spaced closer together and earlier than the ones we used to better assess when differences can first be detected. As is likely apparent, the study just described illustrates another important issue in hormone research: dosing. Future studies on the behavioral effects of hormone supplements may benefit from using multiple levels of exposure rather than using the one we have found useful in our lab [21]. 

An interesting result in the current study is that while TS increased, and CORT and 4-A decreased, time spent in the proximity of a receptive female compared to vehicle-treated animals, TS and vehicle induced similar urinary marking near a receptive female while DHEA, 4A, and CORT supplements led to less marking. Both time spent near a receptive female and urinary marking near a receptive female are established as measures of sexual motivation, but it may be that they differ in mechanism such that they are affected by hormones on a different time course. It is also possible that different levels of hormones are required to affect each behavior such that the levels of TS we administered were enough to increase time spent near a receptive female, but not enough to increase urinary marking. Similarly, it is possible that the levels of DHEA supplements we used were enough to decrease urinary marking near a receptive female, but not enough to decrease time spent near the same female. In this light, DHEA appears to exhibit a unique effect on overall sexual motivation; while DHEA-treated males spent a similar amount of time near estrous females as vehicle-only males, they exhibited significantly less urinary marking than vehicle males in this area. These issues can be clarified in future studies utilizing varying doses and exposure periods.

There are a few important limitations of the current study, some of which have been addressed above. In addition to those already mentioned, a significant limitation of our study is the lack of circulating hormone titers and reproductive organ weight data. Reproductive organ weights would provide a rudimentary measure of hormonal effect on the HPG axis, but circulating hormone measurements would provide definitive support for interpretation of the current results.

There are considerable numbers of reports that 4-A and, particularly, DHEA have other functions than those evaluated here. In the brain, DHEA is a neurosteroid that influences a variety of behaviors, including cognition and mood [22]. Still, it is not clear if neurosteroids must be converted to TS or E2 and bind to the AR and ER receptor because no specific DHEA receptor has been identified. Recently, evidence has emerged that may solve the “DHEA receptor problem.” The suggestion is that the neurosteroids, including DHEA, could influence neural function via interaction with neuronal membranes [23,24,25]. Our results provide evidence that the effects of DHEA and 4-A supplementation are not simply the results of conversion to TS. What seems more likely is that the extra DHEA and 4-A interact directly with neuronal membranes to produce their own sets of behaviors. If confirmed, it would add support for a direct influence of the androgenic adrenal steroids on brain activity.

## 3. Experimental Section

### 3.1. Subjects

Animals (*N* = 100) were experimentally naïve, gonadally intact male (*n* = 60) Long-Evans rats that were 9 weeks of age at the onset of the study. Each had been individually housed for at least 30 days in plastic cages measuring 48.3 cm × 25.4 cm × 20.3 cm with woodchip bedding. Females (*n* = 40) of the same age, strain and source as the experimental males were used as stimulus animals for the behavior tests. Standard rat chow and tap water were available to all animals ad libitum throughout the experiment. Animals were housed in a humidity-controlled colony maintained between 20 °C and 22 °C. Lighting was on a cycle of 12 h light and 12 h dark each day. The Institutional Animal Care and Use Committee of the University of Missouri-St. Louis approved all animal procedures.

### 3.2. Materials

#### 3.2.1. Apparatus

For observations of sexual motivation, we used apparatus and materials described in detail earlier [6,26]. A large Plexiglas terrarium (120 cm × 30 cm × 30 cm) had lines drawn on the outside of the apparatus that divided it into three distinct areas. The two large preference areas on either end of the apparatus measured 52 cm × 30 cm and were separated by a smaller, neutral space measuring 16 cm × 30 cm. In each preference area a stimulus female rat was placed in a wire mesh cage (14 cm × 14 cm × 40 cm), allowing for visual and olfactory investigation by the subject male while limiting physical interactions.

For measurement of urinary marking, sheets of filter paper and a metal screen with openings measuring 0.5 cm^2^ were used [6]. The filter paper, marked with a solid line designating two equal halves, covered the floor of the terrarium underneath a raised wire mesh floor. It was designated as having two equal areas with out a separating neutral space because of the possibility that the male could be marking while his nose and forelimbs were in a preference area and his hindlimbs remained near the center of the apparatus.

Assessment of locomotor activity was conducted on a wooden table with dimensions 122 cm × 91.5 cm. The table received only dim, indirect light. Black lines divide the table’s surface into 15 squares of equal size. A digital timer was used, and squares crossed were tallied using a handheld counter. After each trial, all apparatus were cleaned with a disinfecting and deodorizing antibacterial spray.

#### 3.2.2. Hormones

All hormones were purchased (Sigma Chemical, St. Louis, MO, USA) and suspended in olive oil. Males were subcutaneously injected with either 4-A, DHEA, TS, CORT or oil only (vehicle). Hormones were administered as 400 µg steroid/kg of body weight (bwt) [21]. Estradiol benzoate (100 µg/kg bwt) and progesterone (400 µg/kg bwt) were used to induce behavioral estrus in ovariectomized females [27].

### 3.3. Experimental Design

Males were randomly assigned to 1 of 5 treatment groups (*n* = 12) and SC injected with steroid supplements for 5 weeks. Each animal was behaviorally tested multiple times in the same paradigms. Sexual motivation testing was conducted over the last 3 weeks of supplementation with the result of a 5 × 3 factorial design with the main factor of hormone treatment (4-A, DHEA, TS, CORT or vehicle) and time spent near stimulus female over 3 days of observation as the repeated measure. Urinary marking behavior data and locomotor activity data were summed over the three testing days due to notable variability across days. A 5 × 2 factorial design was used to analyze the urinary marking data, again using hormone treatment as the main factor and number of marks near the stimulus females as the dependent measures. The activity data resulted in a univariate ANOVA with hormone treatment as the main factor.

### 3.4. Procedures

#### 3.4.1. Surgery

Stimulus females were ovariectomized (OVX) under general anesthesia using a single abdominal incision [28]. Uterine horns were exposed and ovaries were excised. All incisions were closed with 3–0 polyglycolic acid sutures. Females were left undisturbed for one week after surgery before hormone treatments were initiated. 

#### 3.4.2. Hormone Administration

Steroids were administered to males six days a week for five weeks. Injections began two weeks prior to testing, and continued for three weeks of behavioral testing. Females were used only as stimulus animals for behavioral measurement of male sexual motivation. All females were OVX, but half (*n* = 20) were induced to estrus with SC injections of estradiol benzoate and, 48 h later, with progesterone.

#### 3.4.3. Sexual Experience

During the two weeks prior to behavioral testing, all males were exposed to a female to gain sexual experience. A female was induced to estrus and placed overnight in the cage of the male on two separate occasions. Observations of copulatory plugs in the bedding the next morning confirmed the animals had copulated. 

#### 3.4.4. Sexual Motivation and Urinary Marking

Sexual motivation was evaluated once a week for three consecutive weeks. On a day in which a male was to be tested for sexual motivation, the apparatus was prepared by placing a stimulus female in each of the two compartments located at the distal ends of the apparatus. One female was OVX and never injected with hormones while the other OVX female was induced to estrus by hormone injections; all females were monitored daily for stage of reproductive cycle by vaginal smear, a procedure described in detail previously [29]. Stimulus females were allowed to acclimate to their compartments for 5 min. Males were then introduced to the neutral area and allowed to explore the entire apparatus for 20 min. The male was determined to show a preference for a stimulus female when his front paws crossed from the neutral area to the preference area in which she was caged. Males were timed in each of the three areas (OVX, OVX-estrous, and neutral). In addition, numbers of urinary marks on the filter paper in the proximity of the estrous female or the non-estrous female were recorded for both halves of the apparatus.

After the 20-min session ended, the subject and stimulus animals were removed to their respective home cages. The filter paper was allowed to dry overnight and then a handheld, long-wave ultraviolet light was used to identify urinary marks. Marks were quantified using a procedure described previously [30]. A sheet of wire mesh was placed over the filter paper, and marks were quantified by counting the number of squares under which urine was detected.

#### 3.4.5. Open Field

On a day that the animal was not tested for sexual motivation, a male was placed at the center of the open field apparatus and allowed to explore for six min. The numbers of squares a subject entered, defined by both hindlimbs fully crossing into the square, were recorded for the six-min trial. Each male was tested for open field activity once a week for three weeks.

#### 3.4.6. Statistical Analyses

The R suite of software facilities from the GNU project was initially used to evaluate univariate normality among the variables. SPSS version 15.0.0 software (SPSS Inc.: Chicago, IL, USA, 2006) for PC was used for the remainder of data analysis. Data analyzed for sexual motivation were time spent in the preference area of the estrous female over the three test sessions and total numbers of urinary markings near either stimulus female. Time spent in the neutral area was not considered in the analyses. Numbers of squares crossed provided the measure of general activity.

A repeated measures 5 × 3 ANOVA was used for time preference data from the sexual motivation apparatus. Main effects were calculated where applicable, and Tukey’s HSD method was used as a post hoc test to compare group means. A repeated measures 5 × 2 ANOVA was used for urinary marks using total marks near the stimulus females as dependent measures; again, Tukey’s HSD method was used for post hoc comparisons. Open field activity was analyzed using a one-way ANOVA using total activity scores across the three sessions. Probability value for all analyses was set at *p* < 0.05.

## 4. Conclusions

In summary, there has been very little published research exploring the effects of such adrenal steroids as a supplement to intact animals. In this study we sought evidence to support or refute the idea that the adrenal steroids simply serve to increase or maintain levels of TS and androgen-sensitive behaviors. Based on the results, it can be concluded that the adrenal supplements 4-A and DHEA leave behavioral “footprints” that are different from that of TS and therefore may be acting directly on the brain rather than serving only as substrates for sex steroids.
